# A New Approach of Sperm Motility Subpopulation Structure in Donkey and Horse

**DOI:** 10.3389/fvets.2021.651477

**Published:** 2021-05-25

**Authors:** Sabrina Gacem, Anthony Valverde, Jaime Catalán, Iván Yánez Ortiz, Carles Soler, Jordi Miró

**Affiliations:** ^1^Equine Reproduction Service, Autonomous University of Barcelona, Department of Animal Medicine and Surgery Veterinary Faculty, Catalonia, Spain; ^2^Costa Rica Institute of Technology, School of Agronomy, Alajuela, Costa Rica; ^3^Universitat de València, Departamento de Biología Celular, Biología Funcional y Antropología Física, Burjassot, Valencia, Spain

**Keywords:** equids, sperm motility, subpopulations, sperm kinematics, CASA-mot

## Abstract

This study aimed to characterize the sperm kinematic values with high frames per second, to define the subpopulation structure of a horse and a donkey and compare them. A total of 57 fresh semen ejaculates (26 Spanish and 16 Arabian horse breeds and 10 donkeys) were collected and subsequently analyzed for kinematic parameters using the Computer-aided sperm motility analysis ISAS®v1.2 system and using a Spermtrack® 10-μm depth counting chamber. Sequences were recorded at 250 frames per second, and eight kinematic parameters were automatically evaluated. All kinematic parameters showed significant differences between a donkey and a horse and between horse breeds. All ejaculates evaluated showed excellent semen motility characteristics, with significantly higher values for all kinematic parameters for donkeys compared with horses except for beat-cross frequency. Donkey sperm was faster and linear than the horse. Regarding horse breeds differences, the Spanish horse had higher average path velocity, curvilinear velocity, and beat-cross frequency compared with the Arabian horse. Spanish horse sperm was rapid, but Arab horse was more linear. The principal component analysis showed three sperm subpopulations in the ejaculate of donkeys and horses with a significantly different motility characteristic between them. The dominant subpopulation for both donkey and horse was for rapid, straight, and linear with a high beat sperm (38.2 and 41.7%, respectively), whereas the lowest subpopulation was for the slowest and non-linear sperms. This, plus slight differences in the distribution of these subpopulations between Arabian and Spanish horses, were found. In conclusion, higher frames permitted to have a new interpretation of motile subpopulations with species and breed differences. More so, future works on donkey and horse breed spermatozoa should take into account differences between breeds that may interfere and alter the real analysis performed.

## Introduction

All the present living species of equids are assigned to the genus *Equus*, sharing a common ancestor million years ago (1), being distributed worldwide in the most diverse environments. The *Equus* genus comprises two lineages: the caballine, which includes domesticated horses (*Equus ferus caballus*), and the wild endangered Przewalski's horse (*Equus ferus przewalskii*) of Mongolia, and non-caballine grouping, which comprises the asses and zebras (2). The domestication process for horses was initiated approximately 450 generations ago, assuming an average generation time of 12 years for wild horses, whereas that for donkeys was around 6,000 years ago (3).

The domestication process started with the need of humans to shape animals' species according to their intentions, producing an artificial selection pressure. The last one has increased particularly since the establishment of studbooks and the development of clear breading objectives affecting the fertility potential (4). Consequently, all the domestic species are now quite far from the original in different aspects involving reproductive characteristics.

The sperm analysis is a prerequisite for breeding soundness examination, and the use of semen in artificial insemination in horses is like in all farm animals. The introduction of computer-assisted semen analysis (CASA) technology allowed for an unprecedented degree of sophistication in the study of sperm characteristics and particularly in reference to kinematics patterns (5, 6). Computer-aided sperm motility analysis (CASA-Mot) systems capture sequences from microscopic fields and automatically analyze all sperm trajectories (7, 8), offering a battery of quantitative kinematic parameters (9).

Improvement in CASA technology and especially the development of new software solutions and new high-performing cameras permitted the authors to analyze better the sperm trajectory (8, 10–12). Recent studies have suggested specific optimum video capture frame rates for a mathematically well-track definition in various species (12, 13). All the previous work using CASA-Mot systems was obtained using suboptimal kinematic data, which resulted in a low significance kinematic parameter. Those are some of the most important limitations in the general use of CASA technology in the past (5).

Furthermore, it was shown that spermatozoa could be grouped into coherent mathematical subpopulations in a wide variety of animals (14–16). These subpopulational structures were also observed for different morphological, biochemical, and physiological traits such as morphometry (17), DNA fragmentation (18), mitochondrial activity (19), osmotic properties (20), and functional status (21). What is clear nowadays is that the ejaculate is not composed of a homogeneous population of equivalent cells but a different subpopulation regarding various cellular properties. Its origin is related both to the testicular formation of the spermatozoa (22) and with their maturational process along the epididymis (23). Interestingly, it can be conditioned by external social interactions, almost in horses (24).

Our study aims to compare the distribution of these kinematic subpopulations between two close equid species horses and donkeys and two horse breeds Spanish and Arabian and that by using higher frames rate for sperm kinematic calculation.

## Materials and Methods

### Animals

The study was conducted at the University Autonoma of Barcelona (Bellaterra, Cerdanyola del Vallès, Spain). Two ejaculates were collected from 13 pure Spanish breeds and eight pure Arabian horses, whereas three ejaculates were obtained from five Catalonian donkeys each, a total of 57 fresh ejaculates. Semen was collected from all animals three times a week throughout the year. All animals ranged from 3 to 15 years of age and fed three times a day with a standard diet (mixed hay and basic concentrate); water was also freely available. All the animals were housed in single boxes in the Equine Reproduction Service of the university. The housing facility is a European Union-approved semen collection center (authorization code: ES09RS01E) that operates under strict protocols of animal welfare and health control. All animals were semen donors and underwent regular semen collection under CEE health conditions (free of equine arteritis, infectious anemia, and contagious metritis). Because this service already runs under the approval of the Regional Government of Catalonia (Spain) and because no manipulation of the animals other than semen collection was carried out, the ethics committee of our institution indicated that no further ethical approval was required.

### Semen Collection

Ejaculates were collected through a pre-warmed artificial vagina model Hannover (Minitüb GmbH, Tiefenbach, Germany) with animals excited by an ovariectomized mare or jenny. An in-line nylon mesh filter was used to separate the gel fraction from the semen. Upon collection, gel-free semen was diluted immediately 1:5 (v:v) in skimmed milk (4.9% glucose, 2.4% skim milk, and 100-mL double-distilled water), previously preheated to 37°C.

Sperm analysis (morphology and concentration) was evaluated upon arrival of semen samples to the laboratory. Sperm concentration was determined using a hemocytometer (Neubauer Chamber; Paul Marienfeld, Germany). To this end, samples were previously diluted with a 4% formalin buffered solution, and the sperm count was adjusted for the dilution factor. Sperm morphology was evaluated by the eosin–nigrosin staining technique.

### Semen Preparation and Computer-Assisted Semen Analysis

The remaining sample was diluted to a final concentration of 40 × 10^6^ spermatozoa/mL, then a volume of 2 μL was mounted on standardized 10-μm depth counting chambers Spermtrack® (Proiser R+D S.L., Paterna, Spain). All chambers were pre-warmed and maintained at 37°C on a UB203 (Proiser R+D)-heated microscope stage throughout the analysis.

Sperm kinematic parameters were automatically assessed using the motility module of CASA system ISAS®v1 (Integrated Sperm Analysis System V1.0; Proiser S.L.; Valencia, Spain). The device is a combination of a Proiser HS640m digital camera mounted on the referred microscope. Images were captured by a 10× negative phase contrast objective (AN 0.25). For each analysis, up to 10 non-consecutive fields were recorded for 3 s at 250 frames per second (fps) in each analysis, permitting the identification of a minimum of 500 spermatozoa per ejaculate.

The settings of the CASA system were those recommended by the manufacturer: particle area >4 and <75 μm^2^; connectivity: 6; cutoff values were VAP ≥ 10 μm/s for a sperm cell to be considered as motile (10, 11). The following sperm motility parameters were determined, sperm velocity: the curvilinear velocity (VCL), straight-line velocity (VSL), and average path velocity (VAP); and sperm movement trajectory: the frequency with which the actual track crossed the smoothed track in either direction [beat-cross frequency (BCF), hertz], and the maximum of the measured width of the head oscillation as the sperm cells swim (the amplitude of lateral head displacement [ALH]). Also, three progression proportions were calculated from the velocity measurements: (the linearity, LIN = VSL/ VCL), the departure of actual sperm track from linearity (wobble WOB = VAP/VCL), and linearity of the average path (straightness, STR = VSL/VAP).

### Statistical Analysis

The data obtained from the analysis of all sperm variables were first tested for normality and homoscedasticity by using Shapiro–Wilks and Levene tests. A normal probability plot was used to check for a normal distribution. Multivariate procedures were performed to identify sperm subpopulations from the set of sperm motility data. All the values for kinematic variables were standardized to avoid any scaling effect.

#### Multivariate Procedures Analysis

Clustering procedures were performed to identify sperm subpopulations from the complete set of motility data. The first step was to perform a principal component analysis (PCA). The number of principal components (PCs) that should be used in the next step of the analysis was determined from the Kaiser criterion, namely selecting only those with an eigenvalue (variance extracted for that PC) > 1. Furthermore, Bartlett's sphericity test and the Kaiser–Meyer–Olkin were performed. As a rotation method, the varimax method with Kaiser normalization was used. The second process was to perform a clustering procedure. A two-step cluster procedure was performed, a hierarchical and a non-hierarchical analysis model, with the sperm-derived indices obtained after the PCA, that uses Euclidean distances from the quantitative variables after standardization of these data, so the cluster centers were the means of the observations assigned to each cluster. In the first step, to determine the optimal number of clusters, the final centroids were clustered hierarchically using the Ward method (25). All sperm cells within different breeds and species were clustered by using the multivariate k-means clustering procedure was made to classify the spermatozoa into a reduced number of subpopulations (clusters) according to their kinematic variables. The clustering procedure enables the identification of sperm subpopulations because each cluster contributed to a final cluster formed by the spermatozoa linked to the centroids. The analysis of variance and χ^2^-test procedures were applied to evaluate statistical differences in the distributions of observations (individual spermatozoa) within subpopulations, and then a generalized linear model procedure was used to determine the effects of the breed and species on the mean kinematic variable values defining the different sperm subpopulations (i.e., the cluster centers). Differences between means were analyzed by the Bonferroni test. Results are presented as mean ± standard error of the mean (SEM). Statistical significance was considered at *P* < 0.05. All data were analyzed using the IBM SPSS package, version 23.0 for Windows (SPSS Inc., Chicago, IL, USA).

## Results

All the kinematic parameters showed significant differences between both species horse and donkey, being higher for donkey unless BCF was higher for the horse. This means that donkey sperm motility was more linear than that of a horse (Table 1).

**Table 1:** Sperm kinematic variables (mean ± SEM) in horses and donkey.

**Variable/Breed**	**Arabian**	**Spanish**	**Total horse**	**Donkey**
VCL	214.52 ± 79.28^a^	232.45 ± 89.55^b^	224.96 ± 85.87^x^	230.08 ± 91.45^y^
VSL	60.14 ± 37.08^a^	59.21 ± 34.83^a^	59.60 ± 35.79^x^	78.07 ± 48.01^y^
VAP	161.59 ± 66.0^a^	167.03 ± 68.89^b^	164.76 ± 67.75^x^	172.63.73 ± 63.08^y^
LIN	26.99 ± 11.95^b^	25.19 ± 11.23^a^	25.94 ± 11.57^x^	33.81 ± 19.26^y^
STR	36.36 ± 15.50^a^	35.49 ± 15.43^a^	35.85 ± 15.46^x^	42.75 ± 19.69^y^
WOB	74.22 ± 9.91^b^	71.21 ± 9.13^a^	72.50 ± 9.58^x^	74.89 ± 14.08^y^
ALH	1.18 ± 0.26^a^	1.30 ± 0.31^b^	1.25 ± 0.30^x^	1.41 ± 0.34^y^
BCF	37.03 ± 15.69^a^	39.25 ± 14.92^b^	38.32 ± 15.29^y^	33.99 ± 18.78^x^

*VCL (μm/s), curvilinear velocity; VSL (μm/s), straight-line velocity; VAP (μm/s), average path velocity; LIN (%), linearity; STR (%), straightness; WOB (%), wobble; ALH (μm), amplitude of lateral head displacement; BCF (Hz), beat-cross frequency. SEM = standard error of the mean*.

a.b *Different superscripts mean significant statistical differences among horse breeds. Different letters (x, y) indicate differences among horse and donkey species P < 0.05*.

When comparing Arabian and Spanish horses, VAP, VCL, ALH, and BCF were higher in Spanish horses than in Arabian, being the contrary for LIN and WOB. VSL, STR, and ALH showed no differences among breeds. Following these results, Spanish horse sperm was faster but less linear than an Arabian horse (Table 1).

The PCA rendered three PCs for both species, explaining 90.9% for stallion and 89.8% for the donkey of the total variance (Table 2). The three PCs were equivalent for donkey and stallion, being PC1, named velocity, positively correlated to the velocity parameters (VCL and VAP) and sperm head oscillation ALH for both species; only BCF was also included for a stallion. PC2, named linearity, was positively correlated to progressivity parameters (LIN and STR) and to VSL. Finally, PC3, named oscillation, was positively correlated to WOB in both species and negatively correlated also to BCF for donkey (Table 2).

**Table 2:** Eigenvectors of the three principal components obtained in the study of sperm kinematics for horse and donkey.

	**Horse**			**Donkey**		
	**PC1**	**PC2**	**PC3**	**PC1**	**PC2**	**PC3**
VCL	0.96			0.86		
VSL		0.79			0.82	
VAP	0.90			0.93		
LIN		0.97			0.89	
STR		0.98			0.98	
WOB			0.97			0.90
ALH	0.90			0.84		
BCF	0.73					−0.67
Explained variation (%)	42.47	32.48	15.96	36.92	32.51	20.40

*PC1, principal component designated “velocity;” PC2, principal component designated “linearity;” PC3, principal component designated “oscillation.” VCL (μm/s), curvilinear velocity; VSL (μm/s), straight line velocity; VAP (μm/s), average path velocity; LIN (%), linearity; STR (%), straightness; WOB (%), wobble; ALH (μm), amplitude of lateral head displacement; BCF (Hz), beat-cross frequency. Only eigenvectors > 0.6 are presented for each principal component*.

**Figure 1:**
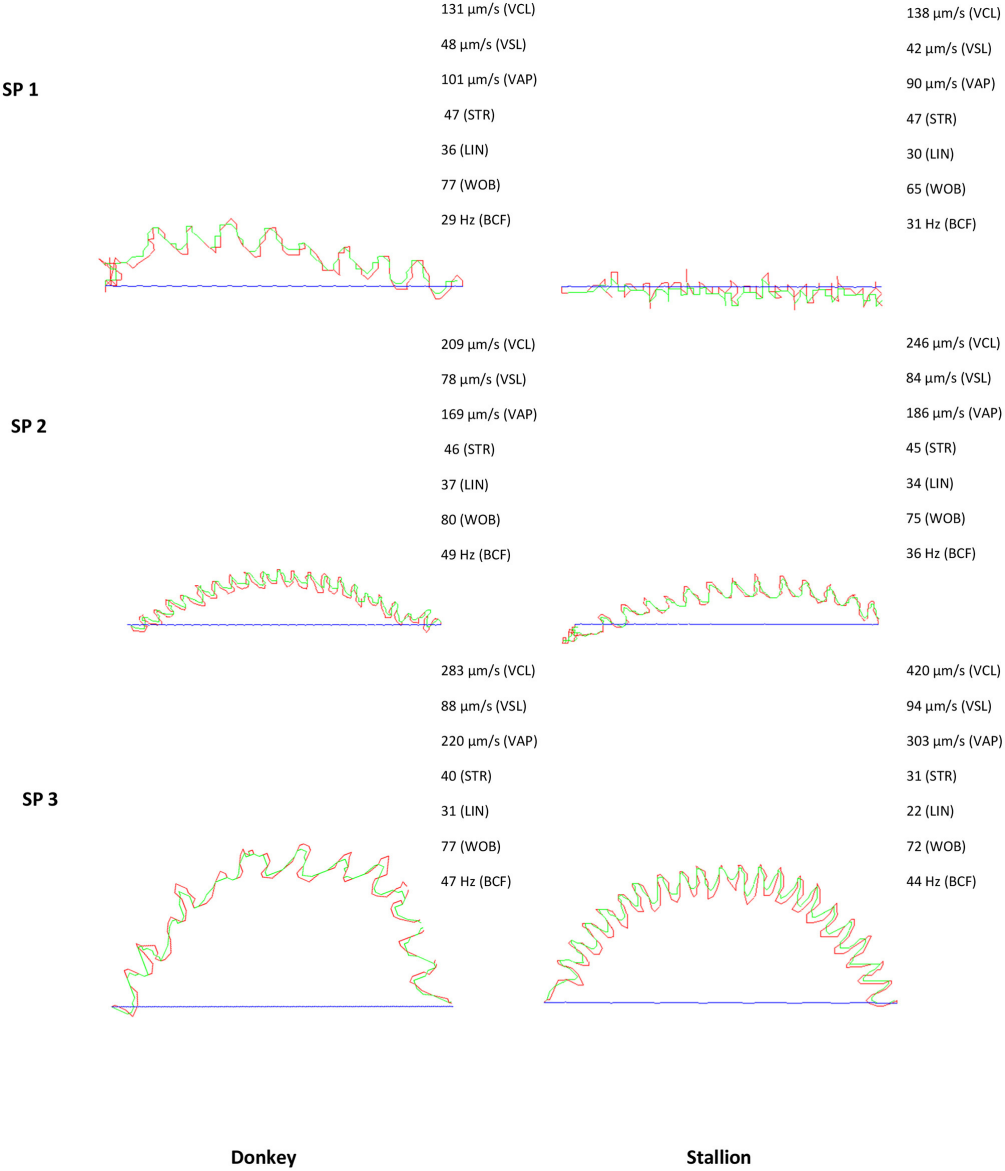
Sperm trajectory of the three subpopulations encountered in stallion and donkey semen SP; sperm subpopulation, Blue line: Straight line velocity (VSL), red line: Curvilinear velocity (VCL); green line: Average path velocity (VAP); straightness (STR); Lineality (LIN); Wobble (WOB); Beat cross frequency (BCF).

These PCs were used to identify three well-defined subpopulations (SP1, SP2, and SP3) in both stallion and donkey, showing differences for all the kinematic parameters among them (Table 3, Figure 1). SP1 had the lowest value of all kinematic parameters, being named as the slow and non-linear subpopulation. SP2 included spermatozoa characterized by the highest linear trajectories (LIN and STR) and high speed (VCL, VSL, and VAP), ALH, and BCF. This subpopulation included the fast, straight, and lineal with a high tail beat spermatozoa subpopulation. SP3 was characterized by the highest VCL, VAP, ALH, and BCF but low linear trajectories (LIN and STR), being defined as the fast with a high beat and non-linear subpopulation (Table 3).

**Table 3:** Descriptive statistics for the CASA-Mot variables (mean ± SD) for each sperm subpopulation species, horse, and donkey samples.

	**Horse**			**Donkey**		
	**SP1**	**SP 2**	**SP 3**	**SP 1**	**SP 2**	**SP 3**
*n*	5,606	7,626	6,714	4,851	7,549	5,678
% sperms	28.1	38.2	33.6	26.8	41.7	31.4
VCL	134.6 ± 48.17^a^	233.45 ± 59.08^b^	290.77 ± 69.08^c^	142.33 ± 47.41^a^	245.8 ± 75.51^b^	286.73 ± 83.72^c^
VSL	26.29 ± 15.67^a^	85.59 ± 30.66^c^	57.89 ± 28.88^b^	26.18 ± 17.15^a^	116.42 ± 32.80^c^	71.41 ± 36.83^b^
VAP	86.18 ± 30.08^a^	176.51 ± 46.0^b^	217.01 ± 49.33^c^	92.65 ± 29.88^a^	191.89 ± 39.25^b^	215.36 ± 45.55^c^
LIN	18.91 ± 8.14^a^	36.6 ± 7.97^c^	19.7 ± 7.80^b^	18.35 ± 10.73^a^	50.29 ± 15.50^c^	25.1 ± 11.01^b^
STR	29.7 ± 13.48^b^	48.63 ± 10.86^c^	26.48 ± 10.94^a^	27.19 ± 14.18^a^	60.67 ± 10.58^c^	32.21 ± 12.96^b^
WOB	64.67 ± 10.08^a^	75.87 ± 7.47^c^	75.21 ± 7.16^b^	66.04 ± 14.83^a^	79.21 ± 14.11^c^	76.69 ± 13.26^b^
ALH	1.03 ± 0.23^a^	1.23 ± 0.22^b^	1.46 ± 0.27^c^	1.08 ± 0.21^a^	1.46 ± 0.25^b^	1.63 ± 0.29^c^
BCF	22.64 ± 10.89^a^	41.85 ± 13.11^b^	47.41 ± 9.88^c^	22.07 ± 11.66^a^	36.8 ± 19.98^b^	40.43 ± 17.39^c^

*n: total number of spermatozoa analyzed; SP1, fast and linear subpopulation; SP2, fast and non-linear subpopulation; SP3, slow and non-linear subpopulation. VCL (μm/s), curvilinear velocity; VSL (μm/s), straight-line velocity; VAP (μm/s), average path velocity; LIN (%), linearity; STR (%), straightness; WOB (%), wobble; ALH (μm), amplitude of lateral head displacement; BCF (Hz), beat-cross frequency. SD = standard deviation*.

a−c *Values with different superscript letters differ significantly between sperm subpopulations of the same species. P < 0.05*.

The percentage of each subpopulation in reference to the total number of spermatozoa varied slightly between donkey and stallion. The stallion and donkey semen have the highest proportion of the fast, straight, and lineal with a high beat subpopulation (SP2: 38.2 and 41.7%, respectively). SP3 was in the middle with 33.6% for stallion and 31.4% for donkey. In contrast, the lowest proportion was for the slow and non-linear subpopulation SP1 (28.2% for stallion and 26.9% for donkey, Table 3).

The proportion of the three stallion subpopulations for Arabian and Spanish horse breeds was almost the same (Table 4).

**Table 4:** Descriptive statistics for the CASA-Mot variables (mean ± SEM) for each sperm subpopulation in two horse breeds, Arabian and Spanish.

	**Arabian**			**Spanish**		
	**SP1**	**SP 2**	**SP 3**	**SP 1**	**SP 2**	**SP 3**
*n*	2,831	3,366	2,128	3,942	4,917	2,762
% sperms	34%	40%	26%	34%	42%	24%
VCL	136.10 ± 47.84^a^	263.81 ± 59.32^c^	240.88 ± 57.11^b^	142.41 ± 45.87^a^	295.39 ± 69.05^c^	248.89 ± 57.97^b^
VSL	30.05 ± 17.47^a^	61.34 ± 26.72^b^	98.25 ± 34.41^c^	31.51 ± 17.57^a^	61.69 ± 29.51^b^	94.35 ± 27.80^c^
VAP	90.61 ± 32.52^a^	206.71 ± 44.26^c^	184.63 ± 45.64^b^	93.47 ± 31.18^a^	219.44 ± 48.70^c^	178.69 ± 40.09^b^
LIN	21.30 ± 9.45^a^	23.04 ± 8.10^b^	40.79 ± 8.82^c^	21.28 ± 8.91^a^	20.99 ± 8.66^a^	38.25 ± 7.76^b^
STR	32.11 ± 14.28^b^	29.25 ± 10.12^a^	53.24 ± 10.85^c^	32.38 ± 13.34^b^	28.14 ± 11.53^a^	53.02 ± 9.68^c^
WOB	66.86 ± 10.12^a^	78.90 ± 6.82^c^	76.89 ± 7.77^b^	65.86 ± 10.01^a^	74.82 ± 6.65^c^	72.42 ± 7.95^b^
ALH	1.02 ± 0.22^a^	1.31 ± 0.23^c^	1.19 ± 0.23^b^	1.05 ± 0.21^b^	1.47 ± 0.28^c^	0.34 ± 0.25^a^
BCF	23.84 ± 11.91^a^	45.33 ± 11.11^c^	41.46 ± 14.81^b^	25.77 ± 11.94^a^	47.39 ± 9.78^c^	44.00 ± 12.79^b^

*n: total number of spermatozoa analyzed; SP1, fast and linear subpopulation; SP2, fast and non-linear subpopulation; SP3, slow and non-linear subpopulation. VCL (μm/s), curvilinear velocity; VSL (μm/s), straight-line velocity; VAP (μm/s), average path velocity; LIN (%), linearity; STR (%), straightness; WOB (%), wobble; ALH (μm), amplitude of lateral head displacement; BCF (Hz), beat-cross frequency. SEM = standard error of the mean*.

a−c *Values with different superscript letters differ significantly between sperm subpopulations of the same breed. P < 0.05*.

## Discussion

Sperm competition plays an important role in sperm fertilization, so in male fertility and lead to a strong natural selection (26). Sperm competition occurs between sperm from two or more rival males making an attempt to fertilize a female within a sufficiently short period (27). This fact has been evidenced not only in mammalian but also in bird (28), fish (29), reptile (30), and insect (31) species. This relevance affects the sperm morphology leading to a production of longer sperms with larger mid-piece (32) and also affecting the head morphometry among close related camelids species (33).

The action of humans along domestication practice implied that natural selection had been replaced by strong artificial selection (34, 35). Domestication of equids took place in the Bronze Age for both horses (36) and donkeys (37). Artificial insemination practice meant a considerable advance in reproduction, and consequently in artificial selection, in all farm animals (38, 39) and recently started in donkey (40). The results presented here showed how close stallion and donkey species are, almost regarding sperm kinematics. In fact, there are more differences between the two considered stallion breeds than between one of them and the donkey. In some mammalian species, like camelids (33), offspring obtained by crossing different species remains fertile, but this is not the case in equids being expected to find higher differences in sperm kinematics. Effectively, the reproductive isolating mechanism is one of the most important speciation processes, and it is frequently related to sperm characteristics variation (41). In equid, species remain very close genetically, being able to produce hybrids viable but not fertile. So, a hinny is a domestic equine hybrid that is the offspring of a male horse (a stallion) and a female donkey (a jenny). It is the reciprocal cross to the more common mule, which is the product of a male donkey (a jack) and a female horse (a mare). Even more, both species can have hybrid offspring with zebras, indicating that evolutive divergency among this species is not enough to avoid reproduction among them.

From the methodological point of view, the results showed in the present work were obtained using the optimal frame rate for both species and stallion breeds (10, 11). Therefore, the subsequent subpopulation analyzed can be considered of high confidence.

Horse and donkey spermatozoa have a different way to move, as the donkey sperm is faster with a more linear trajectory compared with the horse. These differences were also appreciable at a frame rate of 25 fps (42).

To the best of our knowledge, only a few studies compared different horse breeds motility parameters and semen quality (43). Unfortunately, most of those works did not take into consideration kinematic variation between breeds. In the present study, we observed that Spanish breeds present higher sperm velocities (VCL and VAP) than Arab breeds, who showed higher linearity and sperm oscillation.

To complete the classical studies based on the comparison of the median values of each parameter as independent variables, the multivariate statistical procedures, including a reduction of dimensionality by PCA followed by clustering analysis, were developed to define sperm subpopulations (44, 45). During the last years, several studies have shown the universal presence of defined subpopulation structure inside the whole sperm population in the ejaculate (20, 46–48). This fact has changed the previously established paradigm that considered the ejaculate composed of “equivalent” cells competing for reaching the oocyte fertilization. Effectively, some kind of synergies must be present among sperm subpopulations for achieving the final goal of successful fertilization (6, 49).

In the present study, the whole collection of kinematic data was grouped into three PCs in donkey ejaculate, named velocity, progressiveness, and cell oscillation. Using this two-step approach, three subpopulations were obtained, showing that the most frequent SP was for the fastest with high linearity (42% of the total). In a previous study conducted on the Andalusian donkey, four subpopulations were observed, with the main subpopulation (36%) corresponding to low-velocity and high progressive spermatozoa and only 30% corresponding to progressive with high-velocity subpopulation (50). However, it is important to consider that the authors used a CASA-Mot system with only 25 fps, and the statistical procedure was a simple step clustering analysis.

Regarding the horse, the patter bot, both PCs and subpopulations were very similar to that observed in donkey, even if there were significant differences in the kinematic parameters between both species. Again, previous work showed four subpopulations but using a frame rate of just 16 fps (48). Even more, up to six subpopulations were found in other work using a frame rate of 25 fps and following a one-step statistical analysis (46).

All these differences can show the frame rate importance in a correct interpretation of sperm trajectories and the errors that can occur with fewer frames. These changes in sperm trajectories found between different frames also change the real distribution of sperm subpopulations of an ejaculate, all resulting in a misunderstanding of the real role of each subpopulation and its capacity to arrive at the oocyte and fertilize it.

Finally, in reference to the horse breeds considered here, there were no important differences in the sperm subpopulation structure, being little differences in the presence of some of the subpopulations. Instead of this, VCL and VAP for all subpopulations showed significant differences with higher velocities for the Spanish breed compared with Arabian. This could be explained by the history of the domestication of horses who spread out of western central Eurasia, place of origin, that started combined with the continued high genetic input from local wild populations; this hybridization increased genetic differentiation in population, which were accentuated by the human force that shaped their need for creating highly competing horses (1). This hybridization has affected the shape and the performance of the spermatozoa in different degrees depending on the breed, resulting in a decrease in per-cycle conception rates, at around 60% than those observed for other domestic livestock species (51). The Arabian horse is considered one of the most ancestral, with pure pedigree even if it was shown with the recent study of mitochondrial DNA sequences that there is heterogenicity and great diversity among this breed (52), whereas the pure Spanish horse is considered the first European “warmblood,” a mixture of heavy European and lighter Oriental horses, taking its origin from the Andalusia Spanish region that is recognized as a distinct breed since the 15th century (53). Similar differences have been observed in other species like a bull (54), boar (55), and dog (56), showing how much artificial selection procedures conduces to processes close to the speciation process in natural selection (33).

In conclusion, a new performing camera permitted to acquire higher frames for better sperm motility analysis and therefore get more reliable results approachable to real sperm move and changing the old perspectives. The sperm had significantly higher values for all kinematic parameters for the donkey than the horse. Donkey sperm was faster and linear than the horse. Regarding horse breed differences, Spanish horse sperm is rapid, but Arab horse is more linear. The cluster analysis showed three sperm subpopulations; the predominant motile subpopulation in freshly ejaculated horse and donkey sperm had very fast velocity characteristics and a linear trajectory with a high beat frequency. Finally, the identification and differentiation of the structure of functional sperm subpopulations seem to be an advantageous key element as a valuable alternative tool to successfully detect and improve critical handling of further treatment where the effect of the breed would be considered to avoid alterations in the interpretations of the analysis.

Future work is needed to define the relationship of the observed sperm subpopulation structures and the fertility of the samples, considering the effect of different breeds.

## Data Availability Statement

The raw data supporting the conclusions of this article will be made available by the authors, without undue reservation.

## Ethics Statement

Ethical review and approval was not required for the animal study because the ethics committee considered no necessary as explained in the text.

## Author Contributions

CS and JM: conceptualization, validation, visualization, and supervision. SG, JC, IY, and JM: methodology. CS: software. AV: formal analysis. SG, JC, and JM: investigation. SG and CS: data curation. SG and AV: writing—original draft preparation. SG, CS, and JM: writing—review and editing. All authors have read and agreed to the published version of the manuscript.

## Conflict of Interest

The authors declare that the research was conducted in the absence of any commercial or financial relationships that could be construed as a potential conflict of interest.
